# GABAergic and glutamatergic inputs to the medulla oblongata and locus coeruleus noradrenergic pathways are critical for seizures and postictal antinociception neuromodulation

**DOI:** 10.1038/s41598-024-53744-3

**Published:** 2024-02-19

**Authors:** Marcelo Mendonça-dos-Santos, Thaís Cristina Teixeira Gonçalves, Luiz Luciano Falconi-Sobrinho, Tayllon dos Anjos-Garcia, Ivair Matias, Rithiele Cristina de Oliveira, Maria de Fátima dos Santos Sampaio, Fabrízio dos Santos Cardoso, Wagner Ferreira dos Santos, Helio Rubens Machado, Norberto Cysne Coimbra

**Affiliations:** 1 Laboratory of Neuroanatomy and Neuropsychobiology, Department of Pharmacology, Ribeirão Preto Medical School of the University of São Paulo (FMRP-USP), Av. Bandeirantes, 3900, Ribeirão Preto, São Paulo 14049-900 Brazil; 2 Laboratory of Paediatric Neurosurgery and Developmental Neuropathology, Department of Surgery and Anatomy, Ribeirão Preto Medical School of the University of São Paulo, Av. Bandeirantes, 3900, Ribeirão Preto, São Paulo 14049-900 Brazil; 3 Department of Surgery and Anatomy, Multiuser Centre of Neuroelectrophysiology, Ribeirão Preto Medical School of the University of São Paulo (FMRP-USP), Av. Bandeirantes, 3900, Ribeirão Preto, São Paulo 14049-900 Brazil; 4 Neurobiology and Venoms Laboratory, Department of Biology, Ribeirão Preto School of Philosophy, Sciences and Literature of the University of São Paulo (FFCLRP-USP), Ribeirão Preto, SP 14040-901 Brazil; 5 Laboratory of Tissue and Cellular Biology, Centre of Biosciences and Biotechnology of Darcy Ribeiro Northern, Fluminense State University (UENF), Av. Alberto Lamego, 2000, Campos dos Goytacazes, Rio de Janeiro, 28013-602 Brazil

**Keywords:** Refractory epilepsy, Tonic‒clonic seizures, Nucleus of the tractus solitarius, NMDA receptor, GABA_A_ receptor, Locus coeruleus, Noradrenergic pathways, Neuroscience, Anatomy

## Abstract

We investigated the participation of the nucleus of the tractus solitarius (NTS) in tonic‒clonic seizures and postictal antinociception control mediated by NMDA receptors, the role of NTS GABAergic interneurons and noradrenergic pathways from the locus coeruleus (LC) in these phenomena. The NTS-lateral nucleus reticularis paragigantocellularis (lPGi)-LC pathway was studied by evaluating neural tract tracer deposits in the lPGi. NMDA and GABAergic receptors agonists and antagonists were microinjected into the NTS, followed by pharmacologically induced seizures. The effects of LC neurotoxic lesions caused by DSP-4, followed by NTS-NMDA receptor activation, on both tonic‒clonic seizures and postictal antinociception were also investigated. The NTS is connected to lPGi neurons that send outputs to the LC. Glutamatergic vesicles were found on dendrites and perikarya of GABAergic interneurons in the NTS. Both tonic‒clonic seizures and postictal antinociception are partially dependent on glutamatergic-mediated neurotransmission in the NTS of seizing rats in addition to the integrity of the noradrenergic system since NMDA receptor blockade in the NTS and intrathecal administration of DSP-4 decrease the postictal antinociception. The GABA_A_ receptor activation in the NTS decreases both seizure severity and postictal antinociception. These findings suggest that glutamatergic inputs to NTS-GABAergic interneurons, in addition to ascending and descending noradrenergic pathways from the LC, are critical for the control of both seizures and postictal antinociception.

## Introduction

Electrical stimulation of the vagus nerve is a putative treatment for pharmacologically refractory epilepsy, but the neural basis of this procedure is not yet fully understood.

Intraperitoneal (ip) injections of the noncompetitive GABA_A_ receptor antagonist pentylenetetrazole (PTZ) induce tonic‒clonic seizures in laboratory animals The convulsive effect of PTZ is due to GABA-mediated Cl^-^ influx blockade. In addition, although pain sensitivity after PTZ varies according to the age of laboratory animals^1^, seizures caused by PTZ in young adult rodents are followed by significant antinociception^2,3^, a postictal and interictal phenomenon that recruits endogenous opioids^4–8^, norepinephrine^2,9^, serotonin^2,7, 10–13^, and acetylcholine^14–19^. Indeed, neurochemical lesions of brainstem nuclei rich in indolamines^2^, such as the dorsal raphe nucleus; in norepinephrine^2^, such as the locus coeruleus; and in acetylcholine^19^, such as the pedunculopontine tegmental nucleus, impair the postictal antinociception that follows acute tonic‒clonic seizures caused by a single intraperitoneal injection of PTZ.

Electrical stimulation of the vagus nerve activates the nucleus of tractus solitarius (NTS), and several inputs from the vagus nerve through the NTS use glutamate as a neurotransmitter. Reports have shown that the NTS is connected to the nucleus reticularis paragigantocellularis (PGi), a ventromedial medulla oblongata structure also related to the elaboration of at least part of the postictal antinociception^11^.

Although electrical stimulation of the vagus nerve modulates tonic‒clonic seizures^20–22^, possibly through glutamatergic ascending pathways to the NTS, chemical stimulation of the main afferent nucleus of the vagus nerve paradoxically does not necessary contribute to limbic motor seizure modulation^23,24^. There is evidence that an increase in GABAergic signalling or a decrease in glutamate neurotransmission in the NTS reduces susceptibility to tonic clonic seizures^24^, suggesting that the inhibition of NTS efferent pathways can potentially increase the resistance to seizures and provide putative neural bases for the seizure protection obtained with vagal electrical stimulation.

The goal of this work was to investigate the role of the NTS in seizures and postictal antinociception control mediated by NMDA receptors, as well as the role of NTS GABAergic interneurons and noradrenergic pathways from the locus coeruleus (LC) in vagus nerve-related neuromodulation of ictal and postictal syndromes.

## Methods

### Animals

Male Wistar rats (*Rattus norvegicus*, Rodentia, Muridae) weighing 240–250 g (n = 6 per group for neuropharmacological experiments, n = 4 for immunohistochemistry-related procedures, and n = 2 for neural tract tracing experiments; N = 144) from the animal facility of the School of Medicine of Ribeirão Preto of the University of São Paulo (FMRP-USP) were used. The animals were housed 4 to a cage (45 cm × 30 cm × 20 cm) and were habituated to the experimental room for at least 48 h prior to the experiments. During habituation, the animals had free access to water and food. The enclosure was maintained under a light/dark cycle of 12/12 h (lights on from 7 am to 7 pm) and at a constant room temperature of 25 °C ± 1 °C. All the experiments were performed in accordance with the recommendations of the Commission of Ethics in Animal Experimentation of the FFCLRP-USP (proc. 2019.1697591), which are consistent with the ethical principles in animal research adopted by the National Council for Animal Experimentation Control (CONCEA) and were approved by the Commission of Ethics in Animal Research (CETEA) on 17/12/2019. The study was also conducted in accordance with the ARRIVE guidelines.

### Surgical procedure

The animals were anaesthetised with ketamine at 92 mg/kg (Ketamine Agener, União Química Farmacêutica Nacional, São Paulo, Brazil) and xylazine at 9.2 mg/kg (Calmium, União Química Farmacêutica Nacional, São Paulo, Brazil) and fixed in a stereotaxic frame (David Kopf, USA). Stainless steel guide cannulae (outer diameter 0.6 mm, inner diameter 0.4 mm) were implanted in the medial part of the solitary tract nucleus according to the following coordinates: anteroposterior: − 14.04 mm; midline-lateral: 0.4 mm; and dorsoventral: 7.7 mm, targeting 0.6 or 1 mm above the NTS. The coordinates were based on Paxinos and Watson’s rat brain stereotaxic atlas^25^, and the bregma was used as a reference point. The guide cannulae were fixed to the skull using acrylic resin and 2 stainless steel screws. At the end of the surgery, each guide cannula was sealed with a stainless-steel wire to protect it from obstruction. After the surgical procedure, each rodent was treated with an intramuscular injection of penicillin G-Benzatine (120000 UI; 0.1 mL) and a subcutaneous injection of the nonsteroidal analgesic and anti-inflammatory flunixin meglumine (Banamine^®^; 2.5 mg/kg; Schering-Plough, São Paulo, SP, Brazil).

### Nociceptive test

The nociception thresholds were recorded using the tail-flick test. Each animal was placed in a restraining apparatus (Insight, Ribeirão Preto, São Paulo, Brazil) with acrylic walls, and its tail was placed on a heating sensor (tail-flick Analgesia Instrument; Insight). The amount of heat applied to the tail was increased until the animal had its tail removed from the apparatus. The coil (Ni/Cr alloy; 26.04 cm in length × 0.02 cm in diameter) temperature started at room temperature (approximately 20 °C), and then an electric current was applied to increase the temperature of the coil^10,12, 15^. Small adjustments in the current intensity were made, if necessary, at the beginning of the experiment (baseline records) to obtain three consecutive TFLs between 2.5 and 3.5 s. If the animal did not remove its tail from the heater within 6 s, the apparatus was turned off to prevent damage to the skin. Three baseline measurements of control TFLs were made at 5-min intervals. In the experiment, TFLs were measured at 10-min intervals for 180 min immediately after the end of the PTZ-induced seizures (64 mg/kg, ip). The heat was applied to the same portion of the tail (between the middle and distal thirds of the tail of each Wistar rat) at each time point.

### Motor behaviour/experimental seizure test

One week after surgery, the animals were placed in an arena (circular enclosure, 60 cm in diameter and 50 cm high), and the floor was divided into 12 sections. This arena was situated in an experimental compartment illuminated with a 40 W fluorescent lamp (350 lx at the arena floor level). The rats (n = 6 per group) were allowed a 10-min period of habituation in the enclosure at the beginning of each session. The effects of drug administration (PTZ, NMDA, AP7, muscimol, bicuculline, DSP4, and physiological saline) on motor behaviour were subsequently evaluated. PTZ-induced tonic and tonic‒clonic convulsive reactions were used as motor parameters to evaluate the effect of GABA-mediated Cl^-^ influx blockade. The latency of the seizures was defined as the time starting from the injection of PTZ to the first evidence of anterior paw myoclonia^13^. Considering the total duration of convulsive reactions elicited by PTZ at the higher dose used in the present work (an average of 40–60 s) and based on previous works^13,15^, a cut-off of 600 s was used for seizure latency in experiments in which PTZ was administered at lower doses and in the physiological saline-treated groups. The frequency and severity of the seizures were analysed according to Racine’s index^26^, which was subsequently modified by de Freitas et al.^15^ after intraperitoneal (i.p.) administration of PTZ as follows: (a) exploratory behaviour (score 0); (b) jaw and/or facial myoclonic reaction/short duration anterior paw myoclonus (score 1); head myoclonia/moderate myoclonia of the anterior paw with a duration of at least 5 s (score 2); tonic seizures/severe anterior paw myoclonia with a duration of at least 10 s (score 3); tonic seizures/rearing and severe myoclonia of the anterior paw (score 4); tonic seizures/rearing and falling; and myoclonia of the anterior and posterior paws (score 5).

Each animal received a maximum of three treatments. The following experimental groups were performed: (a) PTZ dose‒response curve: Physiological saline (NTS) + saline (i.p.), saline (NTS) + PTZ (34 mg/kg; i.p.), saline (NTS) + PTZ (44 mg/kg; i.p.), saline (NTS) + PTZ (54 mg/kg; i.p.), and saline (NTS) + PTZ (64 mg/kg; i.p.); (b) NMDA receptors activation in NTS: saline (NTS) + saline (i.p.), saline (NTS) + PTZ (64 mg/kg; i.p.), NMDA at 1 nmol (NTS) + PTZ (64 mg/kg; i.p.), NMDA at 3 nmol (NTS) + PTZ (64 mg/kg; i.p.), NMDA at 9 nmol (NTS) + PTZ (64 mg/kg; i.p.); (c) NMDA receptor blockade in NTS: saline (NTS) + saline (i.p.), saline (NTS) + PTZ (64 mg/kg; i.p.), AP-7 at 0.1 nmol (NTS) + PTZ (64 mg/kg; i.p.), AP-7 at 1 nmol (NTS) + PTZ (64 mg/kg; i.p.), AP-7 at 10 nmol (NTS) + PTZ (64 mg/kg; i.p.); (d) Locus coeruleus neurons neurotoxic lesion: saline (i.t.) + saline (NTS) + Saline (i.p.), saline (i.t.) + saline (NTS) + PTZ (64 mg/kg; i.p.), DSP-4 (50 µg; i.t.) + saline (NTS) + PTZ (64 mg/kg; i.p.), DSP-4 (50 µg; i.t.) + NMDA at 9 nmol (NTS) + PTZ (64 mg/kg; i.p.); (e) NTS GABA_A_ receptors activation or blockade on NTS NMDA receptors activation effects: saline (NTS) + saline (NTS) + Saline (i.p.), saline (NTS) + saline (NTS) + PTZ (64 mg/kg; i.p.), muscimol at 250 pmol (NTS) + NMDA at 9 nmol (NTS) + PTZ (64 mg/kg; i.p.), bicuculline at 250 pmol (NTS) + NMDA at 9 nmol (NTS) + PTZ (64 mg/kg; i.p.). The mean and standard error of the mean for each experimental group subjected to different neuropharmacological treatments are provided in Table 1.

**Table 1:** Additional statistical data regarding the neuropharmacological experiments and the mean and standard error of the mean for each experimental group.

Experimental groups	Mean ± S.E.M				
	Index of racine	Seizures (frequency)	Seizures (latency)	Seizures (duration)	Postictal antinociception
Figure 1					
Veh (NTS)/Veh (ip)	0	0	600 ± 0	0	3.059 ± 0.0486
Veh (NTS)/PTZ 34 mg (ip)	1.500 ± 0.2236	1.000 ± 0.2582	27 ± 5.285	9.501 ± 1.045	3.438 ± 0.2756
Veh (NTS)/PTZ 44 mg (ip)	2.000 ± 0.2582	1.333 ± 0.3333	31.50 ± 3.423	36.45 ± 12.79	3.533 ± 0.2608
Veh (NTS)/PTZ 54 mg (ip)	3.000 ± 0.3651	6.667 ± 1.229	39.33 ± 3.676	46.90 ± 14.23	3.939 ± 0.3043
Veh (NTS)/PTZ 64 mg (ip)	4.333 ± 0.3333	10.67 ± 2.667	55.50 ± 2.262	68.49 ± 12.15	4.280 ± 0.3845
Figure 2					
Veh (NTS)/Veh (ip)	0	0	600 ± 0	0	2.728 ± 0.0197
Veh (NTS)/PTZ (ip)	3.822 ± 0.3073	10.67 ± 2.667	50.17 ± 2.428	95.11 ± 2.055	4.306 ± 0.2742
NMDA 1 nmol (NTS)/PTZ (ip)	3.500 ± 0.4282	14.17 ± 2.600	46.33 ± 2.390	58.24 ± 5.191	4.150 ± 0.1572
NMDA 6 nmol (NTS)/PTZ (ip)	3.833 ± 0.1667	11.67 ± 2.578	50.50 ± 3.374	68.54 ± 9.616	3.692 ± 0.1137
NMDA 9 nmol (NTS)/PTZ (ip)	3.500 ± 0.3416	14.33 ± 4.951	105.10 ± 2.364	146.30 ± 9.670	3.191 ± 0.0882
Figure 3					
Veh (NTS)/Veh (ip)	0	0	600 ± 0	0	2.728 ± 0.0196
Veh (NTS)/PTZ (ip)	4.000 ± 0.3651	10.67 ± 2.667	47.83 ± 5.776	42.23 ± 13.69	4.358 ± 0.2893
AP-7 0.1 nmol (NTS)/PTZ (ip)	1.500 ± 0.2236	4.833 ± 0.3073	39 ± 4.583	54.04 ± 29.57	3.539 ± 0.2093
AP-7 1 nmol (NTS)/PTZ (ip)	2.167 ± 0.3073	6.167 ± 0.7032	50.80 ± 4.565	69.24 ± 31.85	2.916 ± 0.0382
AP-7 10 nmol (NTS)/PTZ (ip)	2.000 ± 0.4472	4.000 ± 0.3651	74.80 ± 4.283	19.75 ± 2.516	2.752 ± 0.0199
Figure 4					
Veh/Veh (NTS)/Veh (ip)	0	0	600 ± 0	0	2.993 ± 0.0376
Veh/Veh (NTS)/PTZ (ip)	4.833 ± 0.1667	13.57 ± 1.343	35 ± 2.708	82.90 ± 12.43	4.438 ± 0.2597
Veh/NMDA (NTS)/PTZ (ip)	4.000 ± 0.2582	15.17 ± 4.771	20.33 ± 10.62	161.2 ± 88.19	3.217 ± 0.0990
MUSC/NMDA (NTS)/PTZ (ip)	3.167 ± 0.1667	12.29 ± 1.340	47.83 ± 5.776	31.54 ± 4.30	3.040 ± 0.0342
BIC/NMDA (NTS)/PTZ (ip)	4.333 ± 0.2108	15.00 ± 1.976	48 ± 2.875	143.2 ± 23.98	4.137 ± 0.2126
Figure 5					
Veh (it)/Veh (NTS)/Veh (ip)	0	0	600 ± 0	0	2.692 ± 0.0235
Veh (it)/Veh (NTS)/PTZ (ip)	4.333 ± 0.2108	19.13 ± 2.813	45.17 ± 2.522	142.6 ± 66.24	4.255 ± 0.2706
DSP-4 (it)/Veh (NTS)/PTZ (ip)	3.333 ± 0.5578	16.00 ± 2.366	45.17 ± 7.604	61.51 ± 22.32	2.818 ± 0.0366
DSP-4 (it)/NMDA(NTS)/Veh (ip)	4.333 ± 0.2108	7.833 ± 1.721	52.33 ± 5.897	123.5 ± 93.57	2.826 ± 0.0285

### Drugs

NMDA (N-methyl-D-aspartic acid receptor agonist; Sigma/Aldrich, St. Louis, Missouri, USA) at 1, 3 and 9 nmol/0.2 μL or vehicle (physiological saline; 0.9% NaCl/0.1 μL), the NMDA receptor antagonist AP-7 (2-amino-7-phosphoheptanoic acid) at 0.1, 1, and 10 nmol, the GABA_A_ receptor selective agonist muscimol (5-Aminomethyl-3-hydroxy-isoxazole) in a dose of 250 pmol^24^, the GABA_A_ receptor selective antagonist 1(S),9(R)-(−)-bicuculline methiodide {(5S)-5-[(6R)-6,8-Dihydro-8-oxofuro[3,4-e]-1,3-benzodioxol-6-yl]-5,6,7,8-tetrahydro-6,6-dimethyl-1,3-dioxolo[4,5-g]isoquinolinium iodide} in a dose of 250 pmol^24^, the noradrenergic neurons selective neurotoxin DSP-4 (N-Ethyl-N-(2-chloroethyl)-2-bromobenzylamine hydrochloride)^27^  in a dose of 50 μg, the GABA_A_ receptor channel ionophore blocker pentylenotetrazole (α,β-Cyclopentamethylenetetrazole) at 34, 44, 54, and 64 mg/kg, and physiological saline (0.2 μL) were used in this work. NMDA, AP-7, muscimol, and bicuculline were microinjected into the NTS; DSP-4 was injected intrathecally; and PTZ was administered intraperitoneally. For either the anaesthesia or euthanasia/sacrifice methods, 92 mg/kg ketamine and 9.2 mg/kg xylazine were used before both surgery and brain perfusion. The dose response curve with the N-methyl-D-aspartic acid receptor agonist was performed based on Literature^28^.

### Experimental procedure

In the first set of experiments, the rats received an intraperitoneal (i.p.) injection of PTZ at different doses (34, 44, 54, and 64 mg/kg) to determine the optimal convulsive dose for use in the subsequent experiments. Baseline measurements of the tail-flick test were taken before the drugs were administered, and the tail-flick latencies were also measured immediately after seizures at time 0 and subsequently at 10, 20, 30, 40, 60, 90, 120, 150, and 180 min after the convulsive reactions.

In the second set of experiments, the rats were subjected to stereotaxic surgery to introduce a guide cannula aimed at the NTS. Five days later, baseline measurements of the tail-flick test were taken before unilateral microinjection of either the glutamatergic receptor agonist NMDA (1, 3, or 9 nmol), the NMDA receptor antagonist AP-7 (0.1, 1 or 10 nmol), or physiological saline (0.2 μL) into the NTS, followed by peripheral administration of PTZ at a dose of 64 mg/kg (i.p.) after 5 min.

In the third set of experiments, the effects of the activation or inactivation of GABA_A_ receptors on NTS neurons were investigated via the administration of a single microinjection of muscimol at a dose of 250 pmol, bicuculline at a dose of 250 pmol, or physiological saline (0.2 μL) in NTS, followed by glutamatergic receptor activation via NMDA microinjections in NTS at a dose of 9 nmol, and the peripheral administration of PTZ at 64 mg/kg (i.p.).

In the fourth set of experiments, the role of locus coeruleus noradrenergic neurons in the activation of NTS glutamatergic receptors on seizure severity and postictal antinociception was investigated. For this purpose, rats were pretreated with either a single intrathecal dose of DSP-4 (50 µg; i.t.) or vehicle. After 4 h, the NTS was pretreated with a single microinjection of vehicle or NMDA (9 nmol), followed by peripheral treatment with PTZ (64 mg/kg; i.p.). The combination of NMDA with other drugs in DSP-4-based experiments was performed to comply with the original idea that vagal nerve circuits could be stimulated by the activation of NMDA receptors inside the NTS, a critical brainstem nucleus related to the antiepileptic activity of the vagus nerve electrical stimulation procedure for the treatment of pharmacologically refractory epilepsies.

After each of the procedures described above were performed in independent groups of Wistar rats, the animals were placed in a circular arena for an open-field test for motor behavioural recording for 10 min. Tail-flick latencies were subsequently recorded at 10-min intervals for 180 min.

### Histology

At the conclusion of each experiment, the animals were anaesthetised with ketamine at 92 mg/kg (Ketamina^®^) and xylazine at 9.2 mg/kg (Dopaser^®^) and perfused through the left cardiac ventricle using an infusion pump (Master Flex^®^ L/S TM; Sydney, Australia). The thoracic descending aorta was clamped, the pericardial heart wrap was released to allow perfusion through the left ventricle, and the blood was washed out with Tyrode buffer (40 mL at 4 °C). The animals were then perfused with 200 mL of ice-cold 4% (w/v) paraformaldehyde in 0.1 M sodium phosphate buffer (pH 7.3) for 15 min at a pressure of 50 mmHg. The brain was quickly removed, maintained in 4% paraformaldehyde for at least 4 h and subsequently immersed in a 10% sucrose solution for 48 h. Tissue pieces were immersed in 2-methylbutane (Sigma), frozen on dry ice (30 min), embedded in Tissue Tek (Sakura Finetek, Tokyo, Japan), and cut on a cryostat (Leica CM 1950, Wetzlar, Germany). The sections were then mounted on glass slides that were coated with chrome alum gelatine to prevent detachment and stained in a robotic autostainer (CV 5030 Leica Autostainer) with haematoxylin–eosin. The sections were viewed under a motorised photomicroscope (AxioImager Z1, Carl Zeiss Straβe, Oberkochen, Germany), and the positions of the tips of the guide cannulae were determined according to the Paxinos and Watson's stereotaxic atlas^25^. Data from rats with injector tips located outside the NTS were not included in the statistical analysis.

### Statistical analysis

The data regarding the frequency and severity of the convulsive seizures were subjected to one-way analysis of variance (one-way ANOVA), followed by Newman–Keuls post hoc test. The duration and latency of the seizures were analysed using two-way analysis of variance (two-way ANOVA), followed by Newman–Keuls post hoc test. Data relating to the nociceptive thresholds were subjected to two-way repeated measures analysis of variance (two-way RM ANOVA) using treatment and time as the main factors. In the case of a significant treatment versus time interaction, Newman–Keuls post hoc tests were performed. In all the statistical analyses, the values are reported as the means ± standard errors of the means (S.E.M.), and *P* values < 0.05 were considered to indicate statistical significance.

### Anatomical neural tract tracing

To identify the inputs to PGi and outputs from this nucleus, we unilaterally injected 0.2 μL of a bidirectional neural tract tracer (Alexa Fluor 488-conjugated dextran, 3000 MW; Molecular Probes, Eugene, OR, USA) into the lateral PGi (lPGi) (AP: − 12.48 mm, ML: ± 1.4 mm, and DV: 10.2 mm) of Wistar rats (n = 2). Four days after injection, the mice were anaesthetised and transcardially perfused with 4% paraformaldehyde in phosphate-buffered saline (PBS), after which the encephalon was extracted and processed as previously described^29–31^. The encephalon was cut into 20 µm coronal sections with a cryostat (Leica CM 1950, Wetzlar, Germany). Medulla oblongata slices at the level of the lPGi were used for confirmation of the bidirectional neural tract tracer injection sites, whereas slices from the NTS and LC were used for immunofluorescence staining.

### Immunofluorescence

Immunofluorescence was performed as described elsewhere^31^. Briefly, slices of Wistar rat encephalon (n = 4) were incubated in 0.1 M sodium phosphate buffer (LabSynth, Diadema, São Paulo, Brazil; pH 7.2) overnight. The next day, antigen retrieval with 10 M sodium citrate (pH 6.0) was performed for 30 min in a water bath at 40 °C. The sections were washed three times with 0.1 M sodium phosphate buffer for 5 min each and 0.1 M glycine (Sigma‒Aldrich) for 30 min. The NTS sections were incubated with image-IT (Life Technologies, Carlsbad, CA, USA) for 1 h and simultaneously incubated with the following primary antibodies: mouse monoclonal anti-VGLUT2 (anti-vesicular glutamate transporter 2), IgG (1:1000 dilution; MAB5504, Sigma‒Aldrich), and rabbit polyclonal anti-VGAT (anti-vesicular GABA transporter) IgG (1:500 dilution; cat. 131,002, Synaptic Systems, Göttingen, Germany). The neural tissue sections were washed three times for 5 min each, simultaneously incubated with secondary antibodies (Alexa Fluor 488 goat anti-rabbit IgG 1:1000 and Alexa Fluor 647 goat anti-mouse IgG (1:1000; cat. A-11032; Invitrogen, Carlsbad, CA, USA) for 120 min in the dark, and washed three more times. Finally, the slides were coverslipped with Fluoromount (Thermo Fisher Scientific, Jaguaré, SP, Brazil), and the histological sections were analysed via motorised photomicroscopy (AxioObserver with APOTOME II, Zeiss).

### Ethical approval

We confirm that we have read the journal position on issues involved in ethical publication and affirm that this report is consistent with those guidelines.

## Results

### Effect of increasing doses of PTZ administered intraperitoneally

#### Behavioural seizures

**Figure 1:**
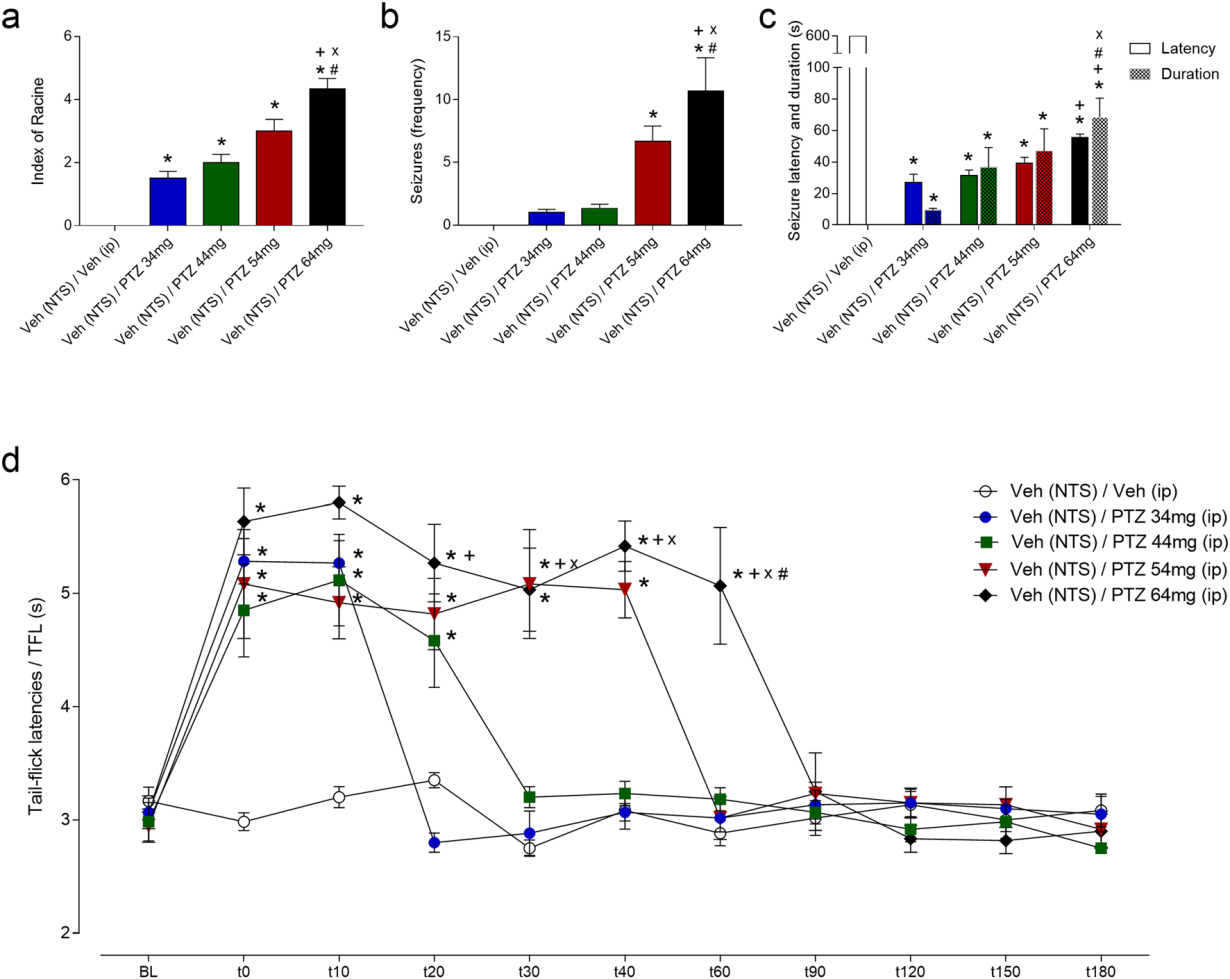
(**a**–**c**) Effect of pretreatment of the nucleus of the tractus solitarius (NTS) with physiological saline followed by intraperitoneal injection of pentylenetetrazole (PTZ) at different doses on the severity (Racine index^26^) (**a**), frequency (**b**) and latency and duration (**c**) of seizures induced by ionophore blockade of GABAergic neurotransmission-related Cl^-^ influx. (**d**): Effect of local NMDA microinjections on the activation of NTS NMDA receptors on the tail withdrawal latency, as recorded by the tail-flick test. *P < 0.05 compared to the control group (vehicle-NTS + vehicle i.p.); ^+^*P* < 0.05 compared to the saline-NTS + PTZ at 34 mg/kg i.p. group; ^x^*P* < 0.05 compared to the saline-NTS + PTZ at 44 mg/kg i.p. group; ^#^*P* < 0.05 compared to the saline-NTS + PTZ at 54 mg/kg i.p. group according to one-way ANOVA (**a**–**b**), two-way ANOVA (**c**), or repeated measures two-way ANOVA (**d**), followed by Neuman–Keuls post hoc test. *BL* tail-flick test baseline latencies; *t* time in seconds.

Intraperitoneal injections of PTZ at different doses preceded by intra-NTS administration of vehicle induced severe tonic‒clonic seizures in the animals. According to one-way ANOVA, compared with those in the vehicle-treated control group, the groups that received intraperitoneal injections of PTZ at 34, 44, 54 and 64 mg/kg preceded by intra-NTS administration exhibited greater seizure severity (index of Racine^26^; F_4,25_ = 36.54; *p* < 0.001), as shown in Fig. 1a. However, compared with those in the vehicle-treated control group, only the animals that received intraperitoneal injections of the highest doses of PTZ (54 and 64 mg/kg) exhibited an increase in seizure frequency (F_4,25_ = 11.88; *p* < 0.001), as shown in Fig. 1b. In addition, the animals that received intraperitoneal injections of PTZ at 64 mg/kg exhibited more seizures, and these convulsive motor reactions were more severe than those in the animal groups that received other doses of PTZ (*p* < 0.05 in all cases), as shown in Fig. 1a,b.

According to the two-way ANOVA, there were significant effects of treatment (F_4,50_ = 483.1; *p* < 0.001) and latency/duration (F_1.50_ = 611.6; *p* < 0.001), as well as an interaction between them (F_4.50_ = 633.7; *p* < 0.001). Compared with those in the control groups, the animals that received intraperitoneal injections of PTZ at different doses exhibited a decreased seizure latency (*p* < 0.05) and an increased seizure duration (*p* < 0.05). In addition, although the animals that received intraperitoneal injections of PTZ at 64 mg/kg exhibited a greater latency, they also exhibited a longer duration of seizures than did the animals that received the other doses of PTZ (*p* < 0.05 in all cases), as shown in Fig. 1c.

### Postictal antinociception

The convulsive motor reactions induced by intraperitoneal injections of PTZ at different doses preceded by intra-NTS administration of vehicle were followed by an increase in tail-flick latencies, a phenomenon known as postictal antinociception, as shown in Fig. 1d.

According to the RM-ANOVA, there were significant effects of treatment (F_4,25_ = 29.56; *p* < 0.001), time (F_10,250_ = 55.19; *p* < 0.001) and the interaction between treatment and time (F_10,250_ = 9.41; *p* < 0.001). The animals that received peripheral injections of PTZ at 34, 44, 54 and 64 mg/kg preceded by intra-NTS administration of vehicle exhibited an increase in tail-flick latencies at 0 to 10, 20, 40, and 60 min, respectively, when compared to the vehicle (NTS) + i.p. vehicle-treated group (*p* < 0.05). In addition, the animals that received intraperitoneal injections of PTZ at 64 mg/kg preceded by intra-NTS administration of vehicle exhibited a greater tail-flick latency from 20 to 60 min than did the animals that received 34 mg/kg PTZ from 30 to 60 min when compared to the animals that received 44 mg/kg PTZ and 60 min when compared to the animals that received 54 mg/kg PTZ, which were preceded by intra-NTS administration of vehicle (*p* < 0.05 in all cases), as shown in Fig. 1d.

### Effect of intra-NTS pretreatment with NMDA at different doses on behavioural seizures and postictal antinociception

#### Behavioural seizures

**Figure 2:**
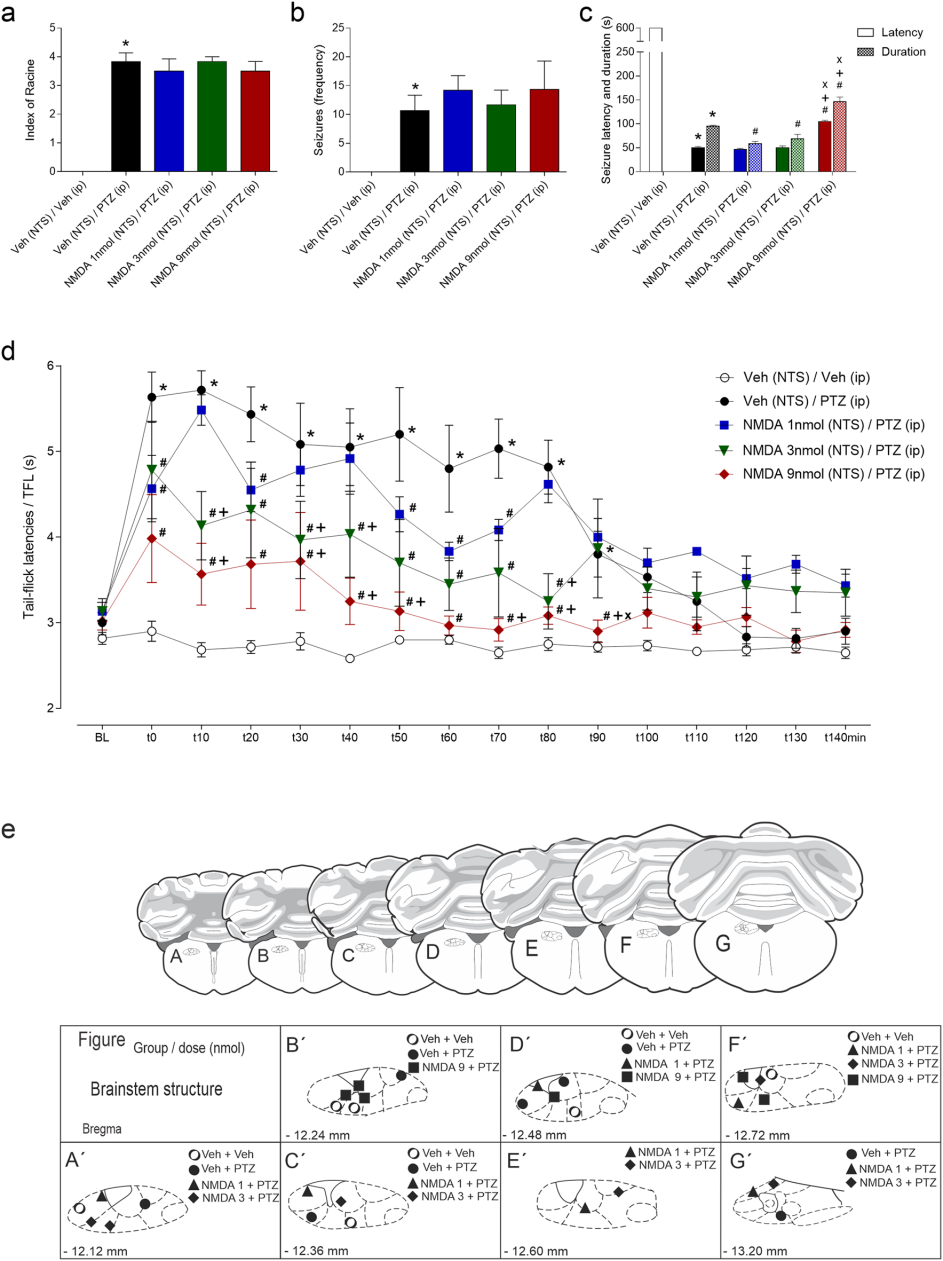
(**a–c**) Effect of glutamatergic receptor activation in the nucleus of the tractus solitarius (NTS) combined with microinjection of NMDA at different doses in the NTS on the severity (Racine index^26^) (**a**), frequency (**b**), and latency and duration (**c**) of seizures induced by pentylenetetrazole (PTZ) injection (64 mg/kg; i.p.). (**d**): Effect of PTZ-induced seizures on tail withdrawal latency recorded by the tail-flick test. *P < 0.05 compared to the control group (vehicle-NTS + vehicle i.p.); ^#^*P* < 0.05 compared to the vehicle-NTS + PTZ at 64 mg/kg i.p. treatment group; ^+^*P* < 0.05 compared to the 1 nmol NMDA-NTS + PTZ at 64 mg/kg i.p. treatment group; and ^x^*P* < 0.05 compared to the 3 nmol NMDA-NTS + PTZ at 64 mg/kg i.p. treatment group according to one-way ANOVA (**a**–**b**), two-way ANOVA (**c**), or repeated measure two-way ANOVA (**d**), followed by Neuman–Keuls post hoc test. (**e**) Schematic representation of the administration of drugs to the nucleus of the tractus solitarius (NTS) according to the following procedures: (**a**) physiological saline (NTS) + saline i.p. (circle), saline (NTS) + PTZ i.p. (filled circle) or NMDA at 1 nmol (NTS) + PTZ i.p. (filled triangle); NMDA at 3 nmol + PTZ i.p. (filled diamond); and NMDA at 9 nmol + PTZ i.p. (filled square), depicted on modified diagrams from the rat brain in the stereotaxic coordinates Atlas by Paxinos and Watson^25^. *BL* tail-flick test baseline latencies; *t* time in seconds.

Intraperitoneal injections of PTZ at 64 mg/kg preceded by intra-NTS administration of vehicle induced severe tonic‒clonic seizures in the animals. According to the one-way ANOVA, compared with the vehicle (NTS) + i.p. vehicle-treated control group, this group exhibited a significant increase in seizure severity (Racine index^26^; F_4,25_ = 32.17; *p* < 0.001) and in the frequency of seizures (F_4,25_ = 3.865; *p* < 0.001). Intra-NTS treatment with NMDA at different doses did not significantly affect seizure severity (index of Racine^26^; *p* > 0.05) or the frequency (*p* > 0.05) of seizures elicited by intraperitoneal injections of PTZ, as shown in Fig. 2a,b.

Regarding seizure latency and duration, there were significant effects of treatment (F_4,50_ = 872.8; *p* < 0.001) and latency/duration (F_1,50_ = 953; *p* < 0.001), as well as an interaction between them (F_4,50_ = 1614; *p* < 0.001), according to two-way ANOVA. The vehicle (NTS) + 64 mg/kg i.p. PTZ-treated group exhibited a significantly longer seizure duration (*p* < 0.05) and shorter seizure latency (*p* < 0.05) than did the vehicle (NTS) + i.p. vehicle-treated group. The animals that received the intra-NTS pretreatment with NMDA at the lowest doses (1 and 3 nmol) exhibited a shorter duration (*p* < 0.05) of seizures than did the vehicle (NTS) + 64 mg/kg i.p. PTZ-treated group. In contrast, the animals that received the intra-NTS pretreatment with NMDA at the highest dose (9 nmol) exhibited longer durations (*p* < 0.05) and latencies (*p* < 0.05) of seizures than did the vehicle + (NTS) + 64 mg/kg i.p. PTZ-treated group and the 1 and 3 nmol NMDA-pretreated groups (*p* < 0.05 in all cases), as shown in Fig. 2c.

#### Postictal antinociception

According to the RM-ANOVA, there were significant effects of treatment (F_4,25_ = 17.9; *p* < 0.001), time (F_15,375_ = 17.64; *p* < 0.001) and the interaction between treatment and time (F_60,375_ = 3.478; *p* < 0.001). Compared with those in the vehicle-treated group, the animals that received peripheral injections of PTZ at 64 mg/kg exhibited an increase in tail-flick latencies from 0 to 90 min (*p* < 0.05). In contrast, compared with those in the i.p. PTZ-treated group, the animals that received intra-NTS pretreatment with NMDA at the highest dose (9 nmol) exhibited a decrease in tail-flick latency in the same time window (*p* < 0.05). In addition, compared to those in the i.p. PTZ-treated group, the animals that received intra-NTS pretreatment with NMDA at the intermediate dose (3 nmol) exhibited a decrease in tail-flick latencies from 0 to 80 min, and the animals that received intra-NTS pretreatment with NMDA at the lowest dose (1 nmol) exhibited a decrease in tail-flick latencies at 0, 20, 50, 60 and 70 min after the end of the seizures (*p* < 0.05 in both cases). When the Wistar rats that received the intra-NTS pretreatment with NMDA were compared, the group that received the highest dose (9 nmol) was significantly different from the animals that received the lowest dose (1 nmol) at 10, 30, 40, 50, 70, 80 and 90 min and different from the animals treated with the intermediate dose (1 nmol) at 90 min postsurgery (*p* < 0.05 in both cases), as shown in Fig. 2d. Histologically confirmed sites of drug microinjection are shown in Fig. 2e.

### Effects of different doses of intra-NTS pretreatment with AP-7 on behavioural seizures and postictal antinociception

#### Behavioural seizures

**Figure 3:**
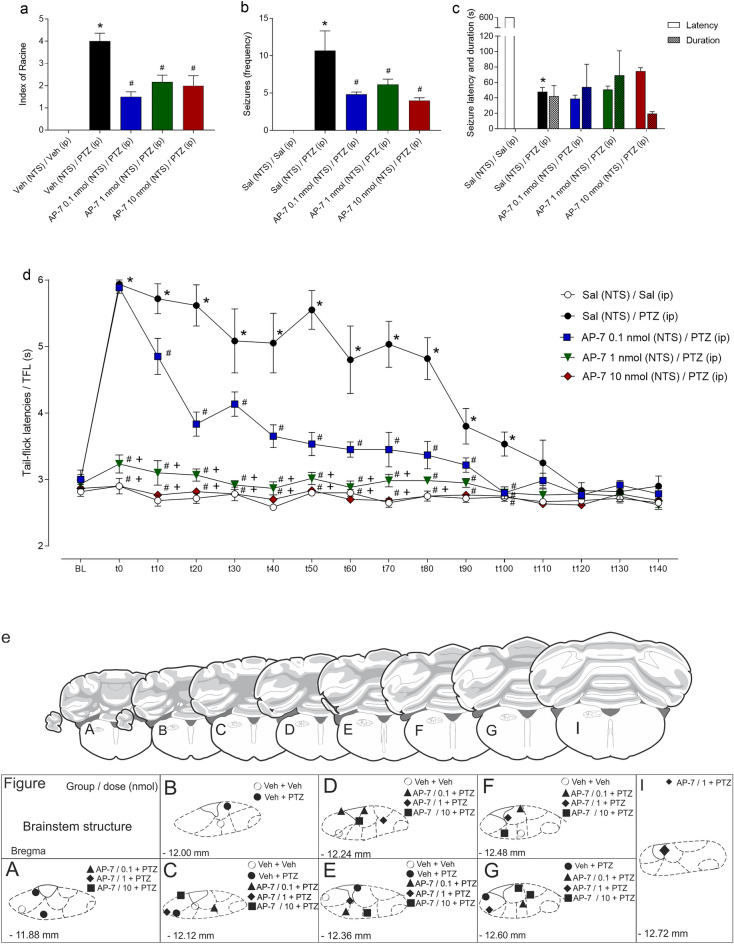
(**a**-**c**) Effect of microinjections of the NMDA receptor-selective antagonist AP-7 at different doses in the nucleus of the tractus solitarius (NTS) on the severity (Racine index^26^) (**a**), frequency (**b**), and latency and duration (**c**) of seizures induced by pentylenetetrazole (PTZ) injection (64 mg/kg; i.p.). (**d**): Effect of PTZ-induced seizures on tail withdrawal latency recorded by the tail-flick test. *P < 0.05 compared to the control group (vehicle-NTS + vehicle i.p.); ^#^*P* < 0.05 compared to the vehicle-NTS + PTZ at 64 mg/kg i.p. treatment group; ^+^*P* < 0.05 compared to the 0.1 nmol AP-7-NTS + PTZ at 64 mg/kg i.p. treatment group according to one-way ANOVA (**a**–**b**), two-way ANOVA (**c**), or repeated measure two-way ANOVA (D), followed by Neuman-Keuls post hoc test. (**e**): Schematic representation of the administration of drugs to the nucleus of the tractus solitarius (NTS) according to the following procedures: (**a**) physiological saline (NTS) + saline i.p. (circle), (b) saline (NTS) + PTZ i.p. (filled circle) or (**c**) AP-7 at 0.1 nmol (NTS) + PTZ i.p. (filled triangle), (**d**) AP-7 at 1 nmol (NTS) + PTZ i.p. (filled diamond), and (**d**) AP-7 at 10 nmol (NTS) + PTZ i.p. (filled square), depicted on modified diagrams from the rat brain in the stereotaxic coordinates Atlas by Paxinos and Watson^25^. *BL* tail-flick test baseline latencies; *t* time in seconds.

The vehicle (NTS) + vehicle (NTS) + PTZ (i.p)-treated group exhibited an increase in seizure severity (index of Racine^26^; F_4,25_ = 21.6; *p* < 0.001) and seizure frequency (F_4,25_ = 9.48; *p* < 0.001) compared to those of the vehicle (NTS) + i.p. vehicle-treated control group according to one-way ANOVA. In contrast, the animals pretreated with Ap-7 at different doses exhibited fewer seizures, and these convulsive motor reactions were less severe than those experienced by the animals that received intraperitoneal injections of PTZ preceded by administration of vehicle into the NTS (*p* < 0.05 in both cases), as shown in Fig. 3a,b.

Regarding seizure latency and duration, there were significant effects of treatment (F_4,48_ = 110.6; *p* < 0.001) and latency/duration (F_1,48_ = 167.8; *p* < 0.001), as well as an interaction between them (F_4,48_ = 156.7; *p* < 0.001), according to two-way ANOVA. The vehicle (NTS) + i.p. PTZ-treated group showed a significant decrease in seizure latency (*p* < 0.05) compared to the vehicle (NTS) + i.p. vehicle-treated group, as shown in Fig. 3c. Intra-NTS pretreatment with AP-7 did not affect the latency (*p* > 0.05) or duration (*p* > 0.05) of seizures elicited by intraperitoneal injections of PTZ, as shown in Fig. 3c.

#### Postictal antinociception

According to the RM-ANOVA, there were significant effects of treatment (F_4,25_ = 154.4; *p* < 0.001), time (F_15,375_ = 35.07; *p* < 0.001) and the interaction between treatment and time (F_60.375_ = 11.85; *p* < 0.001). The vehicle (NTS) + i.p. PTZ-treated group exhibited an increase in tail-flick latency from 0 to 100 min compared to the vehicle (NTS) + i.p. vehicle-treated control group (*p* < 0.05). In contrast, compared with those in the vehicle (NTS) + i.p. PTZ-treated group, the tail-flick latencies of rats subjected to different doses of Ap-7 were lower in the same time window (*p* > 0.05 in both cases). In addition, the groups that received intra-NTS pretreatment with Ap-7 at higher doses (1 and 10 nmol) exhibited a decrease in tail-flick latencies from 0 to 70 and 0 to 80 min, respectively, compared to the 1 nmol (NTS) + i.p. PTZ-treated group (*p* < 0.05 in both cases), as shown in Fig. 3d. Histologically confirmed sites of drug microinjection are shown in Fig. 3e.

### Effects of intra-NTS microinjections of muscimol and bicuculline on behavioural seizures and postictal antinociception

#### Behavioural seizures

**Figure 4:**
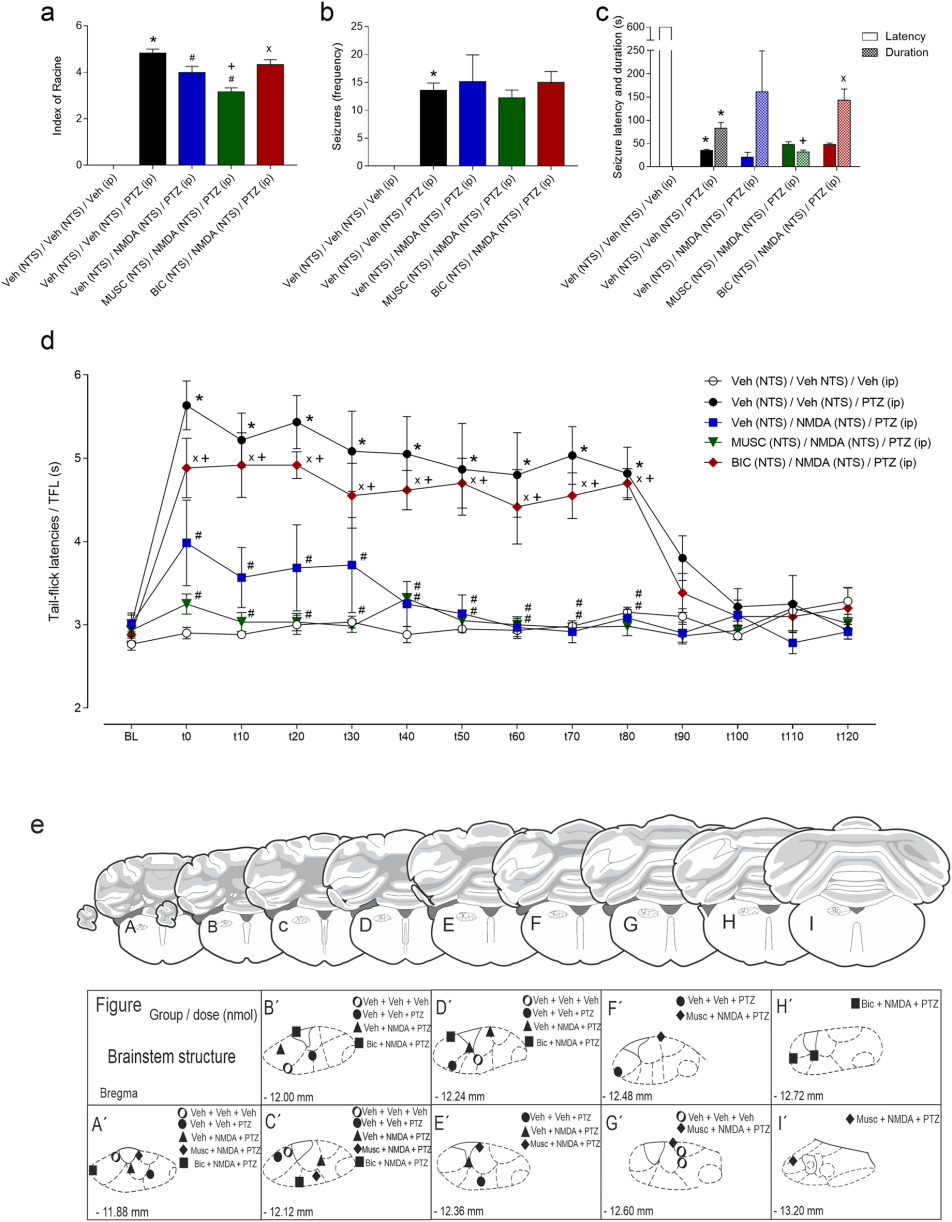
(**a**–**c**) Effect of pretreatment of the nucleus of the tractus solitarius (NTS) with vehicle, muscimol or bicuculline on the effect of glutamatergic receptor activation in the NTS with 9 nmol NMDA on the severity (Racine index^26^) (**a**), frequency (**b**), and latency and duration (**c**) of seizures induced by pentylenetetrazole (PTZ) injection (64 mg/kg; i.p.). (**d**) Effect of PTZ-induced seizures on tail withdrawal latency recorded by the tail-flick test. *P < 0.05 compared to the control group (vehicle-NTS + vehicle-NTS + vehicle i.p.); ^#^*P* < 0.05 compared to the vehicle-NTS + vehicle-NTS + PTZ at 64 mg/kg i.p.)-treated group; ^+^*P* < 0.05 compared to the vehicle-NTS + 9 nmol NMDA-NTS + PTZ at 64 mg/kg i.p.)-treated group; ^x^*P* < 0.05 compared to the muscimol-NTS + 9 nmol NMDA-NTS + PTZ at 64 mg/kg i.p.)-treated group according to one-way ANOVA (A-B), two-way ANOVA (c), or repeated measure two-way ANOVA (**d**), followed by Neuman–Keuls post hoc test. (**e**) Schematic representation of the administration of drugs to the nucleus of the tractus solitarius (NTS) according to the following procedures: (**a**) physiological saline (NTS) + saline (NTS) + saline ip (circle), (**b**) saline (NTS) + saline (NTS) + PTZ i.p. (filled circle) or (**c**) saline (NTS) + NMDA (NTS) + PTZ i.p. (filled triangle), (**d**) muscimol (NTS) + NMDA (NTS) + PTZ i.p. (filled diamond), and (**e**) bicuculline (NTS) + NMDA (NTS) + PTZ i.p. (filled square), depicted on modified diagrams from the rat brain in the stereotaxic coordinates Atlas by Paxinos and Watson^25^. *BL* tail-flick test baseline latencies; *t* time in seconds.

The vehicle (NTS) + vehicle (NTS) + PTZ (i.p.)-treated group exhibited an increase in seizure severity (index of Racine^26^; F_4,25_ = 111.1; *p* < 0.001) and seizure frequency (F_4,29_ = 8.142; *p* < 0.001) compared to those of the vehicle (NTS) + vehicle (NTS) + i.p. vehicle-treated control group according to one-way ANOVA, as shown in Fig. 4a,b. The vehicle (NTS) + NMDA (NTS) + i.p. PTZ- and MUSC (NTS) + NMDA (NTS) + i.p. PTZ-treated groups exhibited a decrease in seizure severity compared to the vehicle (NTS) + vehicle (NTS) + i.p. PTZ-treated group (*p* < 0.05). In addition, the seizure severity after MUSC + NMDA + PTZ treatment was significantly lower than that displayed by Veh + NMDA + PTZ-treated rats and when compared to BIC + NMDA + PTZ-treated group reactions. These data are shown in Fig. 4a.

Regarding seizure latency and duration, there were significant effects of treatment (F_4,50_ = 25,32; *p* < 0.001) and latency/duration (F_1,50_ = 12.71; *p* < 0.001), as well as an interaction between them (F_4,50_ = 53.16; *p* < 0.001), according to two-way ANOVA. The vehicle (NTS) + vehicle (NTS) + i.p. PTZ-treated group exhibited significantly greater seizure latencies (*p* < 0.05) and durations (*p* < 0.05) than did the vehicle (NTS) + vehicle (NTS) + i.p. vehicle-treated control group, as shown in Fig. 4c. The vehicle (NTS) + NMDA (NTS) + i.p. PTZ-treated group was not different from the vehicle (NTS) + vehicle (NTS) + PTZ (i.p.)-treated group in terms of both seizure latency (*p* > 0.05) and duration (*p* > 0.05). However, the MUSC (NTS) + NMDA (NTS) + i.p. PTZ-treated group exhibited a shorter duration of seizures than the vehicle (NTS) + NMDA (NTS) + i.p. PTZ-treated group and the BIC (NTS) + NMDA (NTS) + i.p. PTZ-treated group exhibited a longer duration of seizures than the MUSC (NTS) + NMDA (NTS) + i.p. PTZ-treated group did (*p* < 0.05 in both cases), as shown in Fig. 4c.

#### Postictal antinociception

According to the RM-ANOVA, there were significant effects of treatment (F_4,25_ = 38.43; *p* < 0.001), time (F_13,325_ = 15.54; *p* < 0.001) and the interaction between treatment and time (F_52,325_ = 4.281; *p* < 0.001). The vehicle (NTS) + vehicle (NTS) + i.p. PTZ-treated group exhibited an increase in tail-flick latencies from 0 to 80 min compared to the vehicle (NTS) + vehicle (NTS) + i.p. vehicle-treated control group (*p* < 0.05). In contrast, both the vehicle (NTS) + NMDA (NTS) + i.p. PTZ-treated group and the MUSC (NTS) + NMDA (NTS) + i.p. PTZ-treated group exhibited significantly shorter tail-flick latencies in the same time window than did the vehicle (NTS) + vehicle (NTS) + i.p. PTZ-treated group (Newman–Keuls post hoc test;* p* < 0.05 for both cases), as shown in Fig. 4d. However, compared with vehicle (NTS) + NMDA (NTS) + i.p., PTZ treatment did not significantly decrease postictal antinociception but did significantly differ from the vehicle (NTS) + NMDA (NTS) + i.p. PTZ-treated group, as shown in Fig. 4d, possibly due to the increase in the duration of seizures caused by pretreatment of the NTS with bicuculline in comparison to that caused by pretreatment of the NTS with muscimol, as shown in Fig. 4c. Histologically confirmed sites of drug microinjection are shown in Fig. 4e.

### Effect of intrathecal microinjections of DSP-4 on behavioural seizures and postictal antinociception

#### Behavioural seizures

**Figure 5:**
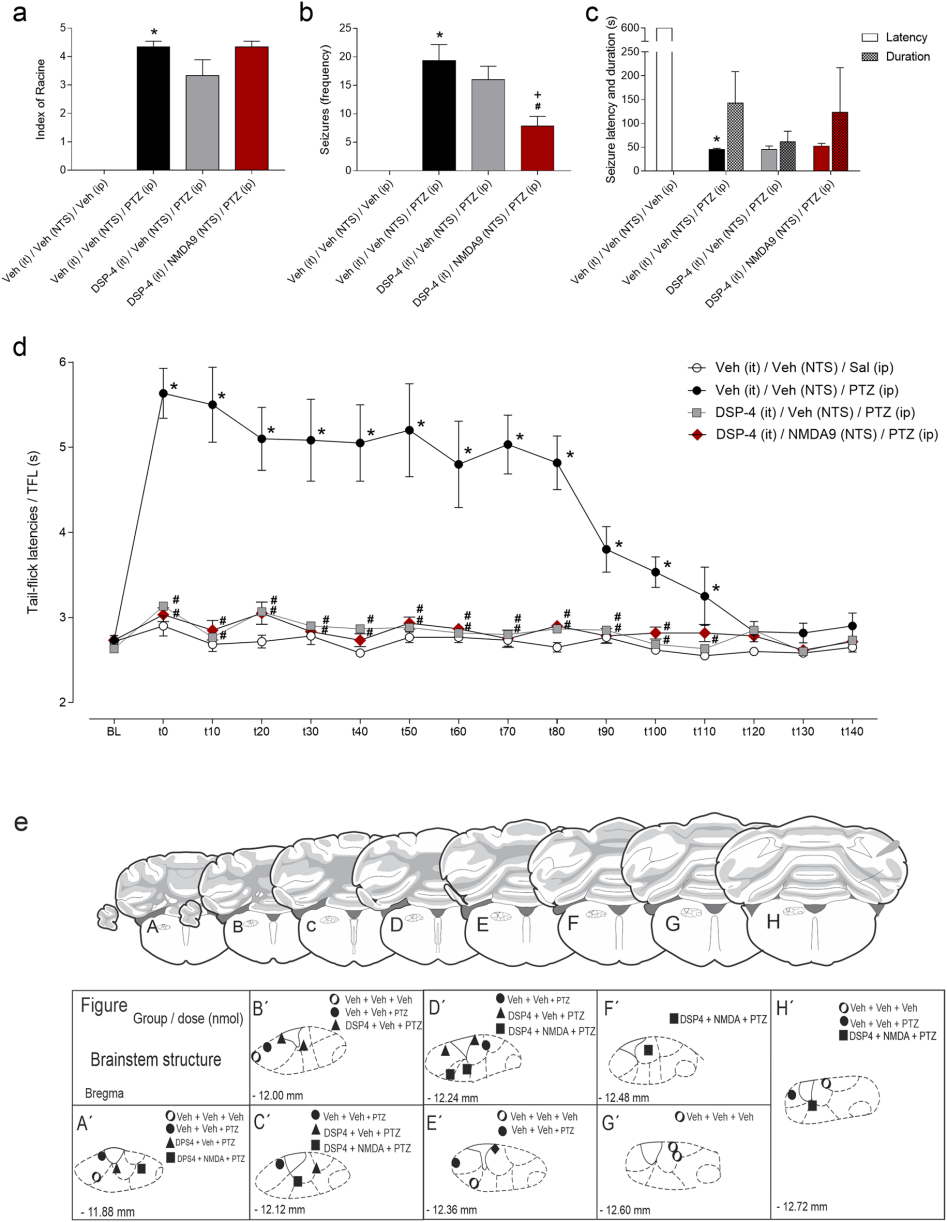
(**a**–**c**) Effect of pretreatment with a single intrathecal microinjection of DSP-4 (50 µg/0.2 µL) on the effect of glutamatergic receptor activation in the nucleus of the tractus solitarius (NTS) with 9 nmol NMDA on the severity (Racine index^26^) (**a**), frequency (**b**), and latency and duration (**c**) of seizures induced by pentylenetetrazole (PTZ) injection (64 mg/kg; i.p.). (**d**) Effect of PTZ-induced seizures on tail withdrawal latency recorded by the tail-flick test. *P < 0.05 compared to the control group (vehicle-i.t. + vehicle-NTS + vehicle i.p.); ^#^*P* < 0.05 compared to the vehicle i.t. + vehicle-NTS + PTZ at 64 mg/kg i.p.)-treated group according to one-way ANOVA (**a**–**b**), two-way ANOVA (**c**), or repeated measures two-way ANOVA (**d**), followed by Neuman–Keuls post hoc test. (**e**): Schematic representation of the administration of drugs to the nucleus of the tractus solitarius (NTS) according to the following procedures: (**a**) physiological saline (it) + saline (NTS) + saline i.p. (circle), (**b**) saline (i.t.) + saline (NTS) + PTZ i.p. (filled circle) or (**c**) DSP-4 (i.t.) + saline (NTS) + PTZ i.p. (filled triangle), and (**d**) DSP-4 (i.t.) + NMDA at 9 nmol (NTS) + PTZ i.p. (filled square), depicted on modified diagrams from the rat brain in stereotaxic coordinates Atlas by Paxinos and Watson^25^. BL: tail-flick test baseline latencies; t: time in seconds.

Intraperitoneal injections of PTZ at 64 mg/kg preceded by intra-NTS microinjections of vehicle or intrathecal administration of vehicle induced severe tonic‒clonic seizures in the experimental animals. According to the one-way ANOVA, compared with the i.t. vehicle + vehicle (NTS) + i.p. vehicle-treated control group, the experimental group exhibited an increase in seizure severity (Racine index^26^; F_3,20_ = 42.22; *p* < 0.001) and seizure frequency (F_3,20_ = 18.24; *p* < 0.001), as shown in Fig. 5a,b. Compared with those in the i.t. vehicle + vehicle (NTS) + i.p. PTZ-treated group, the animals in the group that received intrathecal DSP-4 followed by microinjections of NMDA in the same medulla oblongata structure and intraperitoneal administration of PTZ exhibited a decreased seizure frequency (*p* > 0.05). Treatment with DSP-4 + NMDA (9 nmol + PTZ) caused a significantly lower frequency of seizures than did DSP-4 + Veh + PTZ treatment alone. These data are shown in Fig. 5b.

Regarding seizure latency and duration, there were significant effects of treatment (F_3,40_ = 14.67; *p* < 0.001) and latency/duration (F_1,40_ = 12.54; *p* < 0.001), as well as an interaction between them (F_3,40_ = 32.2; *p* < 0.001), according to two-way ANOVA. The i.t. vehicle + vehicle (NTS) + i.p. PTZ-treated group showed a significant decrease in seizure latency (*p* < 0.05) compared to the i.t. vehicle + vehicle (NTS) + i.p. vehicle-treated group, as shown in Fig. 5c. Intrathecal DSP-4 pretreatment with DSP-4 did not affect the latency (index of Racine^26^; *p* > 0.05) or duration (*p* > 0.05) of seizures elicited by intraperitoneal injections of PTZ, as shown in Fig. 5c.

#### Postictal antinociception

According to the RM-ANOVA, there were significant effects of treatment (F_3,20_ = 223; *p* < 0.001), time (F_15,300_ = 13.1; *p* < 0.001) and the interaction between treatment and time (F_45.300_ = 7.563; *p* < 0.001). The i.t. vehicle + vehicle (NTS) + i.p. PTZ-treated group exhibited an increase in tail-flick latencies from 0 to 110 min when compared to the i.t. vehicle + vehicle (NTS) + i.p. vehicle-treated control group (*p* < 0.05). In contrast, both the DSP-4 (i.t.) + vehicle (NTS) + i.p. PTZ-treated and i.t. DSP-4 + NMDA (NTS) + i.p. PTZ-treated groups exhibited a decrease in tail-flick latencies in the same time window compared to the i.t. vehicle + vehicle (NTS) + i.p. PTZ-treated group (*p* < 0.05 in both cases), as shown in Fig. 5d. Histologically confirmed sites of drug microinjection are shown in Fig. 5e.

### Interactions between glutamatergic inputs to NTS GABAergic interneurons and the NTS-lPGi-LC pathway

**Figure 6:**
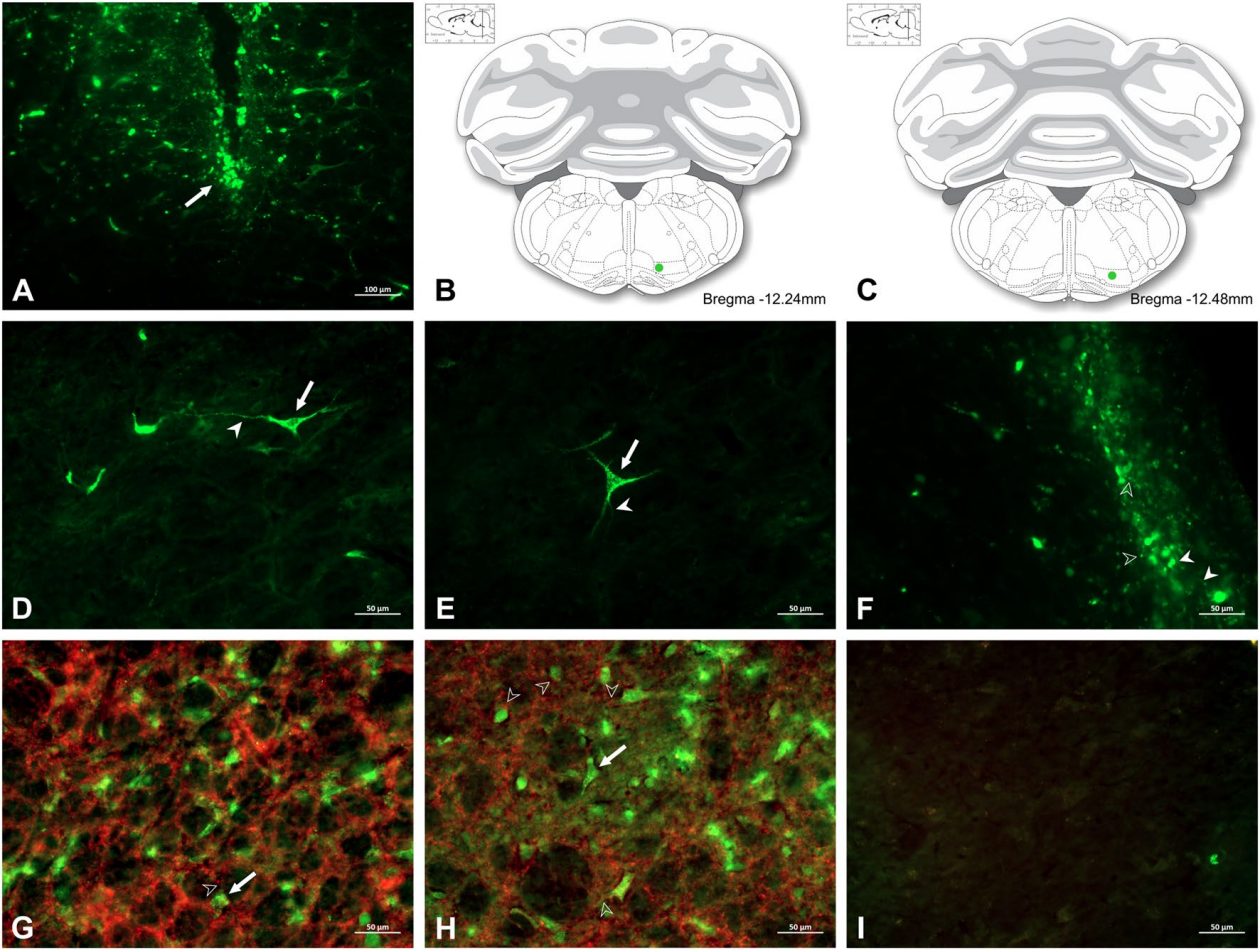
(**a**–**c**) Photomicrograph of transverse section of lateral paragantocellularis nucleus (lPGi), showing a representative site (white arrow) of microinjection of AlexaFluor 488-conjugated dextran neural tract tracer (**a**) and drawings of transverse sections of medulla oblongata (**b**, **c**) showing histologically confirmed sites (green circles) of neural tract tracer deposits in lPGi. (**d**–**f**) Photomicrographs of transverse sections of the medulla oblongata (**d**, **e**) and pons (**f**) showing AlexaFluor-488-conjugated dextran-labelled neurons (white arrows) and neuronal fibres (closed arrowheads) situated in the nucleus of tractus solitarius (NTS), in addition to neurotracer-labelled fibres (closed arrowheads) and terminal buttons (open arrowheads) in the locus coeruleus (**f**). (**g**–**i**) Photomicrographs of NTS transverse sections showing AlexaFluor 647-VGLUT-labelled glutamatergic appositions (open arrowheads) on AlexaFluor 488-VGAT-labelled dendrites and perikarya (white arrows) of GABAergic neurons situated in the NTS of Wistar rats. As a control, NTS tissue was processed without the use of each primary antibody (**i**).

We performed a double immunolabelling of VGLUT and VGAT fibres and perikarya in NTS to identify the glutamatergic inputs from the vagus nerve to GABAergic neurons situated in the NTS, as well as to characterise the NTS- lPGi-LC pathway. In an independent experiment, we microinjected the LPGi cells with AlexaFluor 488-conjugated dextran, as shown in Fig. 6a–c. The neural tract tracer retrograde-filled AlexaFluor488-conjugated dextran-labelled perikarya in the NTS (Fig. 6d,e) indicated the NTS-lPGi pathway. We also found neural tract tracer-labelled fibres in in the LC, indicating that outputs from lPGi reach LC neurons (Fig. 6f). VGLUT-labelled neuronal appositions were also found throughout the NTS (Fig. 6g,h), reaching dendrites and perikarya of VGAT-labelled neurons in the NTS (Fig. 6g,h), suggesting glutamatergic inputs from the vagus nerve to NTS GABAergic interneurons. For the control groups, primary antibodies were omitted, as shown in Fig. 6i (representative photomicrograph).

## Discussion

The ionophore blockade of GABA_A_ receptor-related chloride channels caused by PTZ injection caused tonic‒clonic seizures followed by postictal antinociception in all animals included in the present study. The severity of both pharmacologically induced seizures and postictal antinociception was shown to be partially dependent on the activity of excitatory amino acid-mediated pathways connected to the NTS and on the integrity of the noradrenergic system since the blockade of NMDA excitatory amino acid receptors in the NTS and the intrathecal administration of a neurotoxin selective for noradrenergic neurons decreased the severity of tonic‒clonic seizures and the intensity of postictal analgesia. There is evidence that the noradrenergic system is involved in the control of cortical activity^32–34^. The locus coeruleus is an eminently noradrenergic structure that sends ascending connections that are distributed within the neocortex^34^ and descending connections modulated by dorsal midbrain structures^2^ that reach the dorsal horn of the spinal cord, modulating the activity of the first synapse of the ascending somatosensory discriminative pain pathways.

The locus coeruleus is a relevant structure of the brainstem that is also involved in the antiepileptic activity caused by stimulation of the vagus nerve^35,36^. The general visceral afferent fibres (parasympathetic afferents) and the special visceral afferent fibres (gustatory fibres that innervate the epiglottis) of neurons from the vagus nerve (lower) ganglion project to the caudal and cranial division of the NTS, and glutamate is the main neurotransmitter^37–39^. We showed that the NTS, in turn, is also connected to the brainstem lateral paragigantocellular reticular nucleus, whose neurons project to the locus coeruleus. These findings corroborate previous reports suggesting that NTS neurons, in addition to connecting with the dorsal thalamus through the trigeminal lemniscus, also send outputs to lPGi that are connected to the LC^40,41^, although these former projections should be considered with caution, considering that we cannot precisely identify terminal buttons close to LC neuronal bodies. Thus, it was expected that the activation of glutamatergic receptors in the NTS would cause an anticonvulsant effect.

However, as demonstrated in the present work, microinjection of a glutamatergic agonist selective for NMDA receptors into the NTS at different doses did not significantly alter the severity of seizures nor its frequency, latency or duration of seizures, although the activation of NMDA receptors in the NTS significantly decreased postictal antinociception. A possible explanation for this phenomenon could be the presence of inhibitory interneurons between the afferents of the primary neuron of the ganglion nodosum (the inferior vagal nerve ganglion) and the NTS neurons or inhibitory projections from other brainstem nuclei connected to the NTS. In fact, we demonstrated that VGLUT-labelled appositions are found on dendrites and perikarya of GABAergic neurons in the NTS and in a profuse GABAergic neural network surrounding NTS neurons. These morphological findings support the hypothesis that vagus nerve glutamatergic afferent pathways recruit GABAergic interneurons to the NTS or GABAergic terminals from other brainstem structures connected to the NTS to accomplish the anticonvulsant effects of vagus nerve stimulation. Indeed, there are additional reports that GABAergic neurons can be abundantly found throughout the NTS^42^. In addition, NTS neurons may receive both glutamatergic and GABAergic afferents from local neurons, which may be activated by afferent nerve inputs or modulated by afferents from other brainstem neurons^43^. Interestingly, blockade of NMDA receptors in the NTS significantly reduced both the severity of seizures and the frequency of seizures and significantly reduced postictal antinociception, as demonstrated in our work. These data corroborate a previous report in which microinjection of the nonselective antagonist of excitatory amino acid receptors in the NTS, cinurenic acid, was shown to cause an anticonvulsive effect^24^. Interestingly, desensitisation of AMPA receptors impairs the duration and severity of seizures, and failure of this mechanism predisposes patients to severe and prolonged seizures^44^. In addition, increases in GABAergic neurotransmission or a decrease in glutamate transmission in the NTS reduce susceptibility to limbic seizures in a pharmacologically induced epilepsy model^24^. Interestingly, we demonstrated that pretreatment of the NTS with the GABA_A_ receptor selective agonist muscimol, rather than pretreatment of the NTS with the selective GABA_A_ receptor selective antagonist bicuculline followed by chemical activation of the NTS with NMDA, decreased the severity of PTZ-induced seizures, in addition to completely significantly blocking postictal antinociception. These findings highlight the relevance of GABA-signalling-related neurochemical mechanisms in the NTS for the control of pharmacologically induced tonic clonic seizures and postictal antinociception.

Pretreatment of the NTS with the GABA_A_ receptor agonist muscimol at a dose of 250 pmol but not with the GABA_A_ receptor bicuculline at the same dose, followed by NMDA (9 nmol) microinjections in the NTS, decreases the severity of tonic‒clonic seizures, suggesting an anticonvulsant effect on the activation of GABA_A_ receptors in the NTS. A decrease in the severity of seizures was also followed by a significant decrease in postictal antinociception. We hypothesised that the increase in activity of GABAergic interneurons in the NTS could cause impairment of a possible inhibitory pathway from the NTS to the lPGi and that the consequent augmented activity in lPGi neurons could activate LC ascending noradrenergic pathways, causing an anticonvulsant effect.

Indeed, neural tract tracer deposits in the lPGi showed neurotracer-labelled fibres in the LC. Interestingly, according to the present study, the neurotoxic damage caused by DSP-4, a neurotoxin that competes with the substrate of the beta-hydroxylase enzyme and has particular tropism for locus coeruleus neurons^27^, decreases both the frequency of tonic seizures and postictal antinociception.

Therefore, the effect of intrathecal injection of DSP-4, which is associated with the activation of NTS-NMDA receptors, may be due to one of the following neural mechanisms: (a) impairment of direct activation of the locus coeruleus by NTS neurons that eminently activate neurons related to noradrenergic ascending pathways that result in modulation of neocortical activity; or (b) decreased activity of the noradrenergic descending antinociceptive pathway due to depletion of noradrenaline in the locus coeruleus caused by DSP-4. Indeed, norepinephrine is a key neurotransmitter involved in the regulation of excitability and plasticity of large-scale brain networks; for example, it has been shown to exert a modulatory effect on basal synaptic transmission in the dorsal hippocampus and enhance basal neuronal excitation in the dorsal hippocampus rather than the ventral hippocampus^45^, a relevant structure damaged in temporal lobe epilepsy. There is also evidence that norepinephrine transporter antagonism prevents dopamine-dependent late long-term synaptic potentiation (LTP) in the dorsal hippocampus of mice^46^. In addition, despite evidence for both the inhibition and excitation of LC neurons during seizures, a consistent release of noradrenaline in seizing animals submitted to a temporal lobe epilepsy experimental model was recently reported^47^, and interestingly, concentrations of norepinephrine increased at seizure onset and decreased during or shortly after the seizure. In addition, there is evidence that β-norepinephrinergic receptor-mediated LTP is strongly reduced in the distal subiculum of laboratory animals subjected to a pilocarpine model of epilepsy^48^. Moreover, monoaminergic neurotransmitters/neuromodulators were demonstrated by our team to play a key role in postictal antinociception organisation^9^. The α_2_-norepinephrinergic receptor selective antagonist yohimbine and the β-norepinephrinergic receptor nonselective antagonist propranolol were microinjected unilaterally into the locus coeruleus, followed by intraperitoneal administration of PTZ, which resulted in a significant decrease in tonic or tonic‒clonic seizure-induced antinociception.

Taken together, these data suggest that the anticonvulsive effect of increased glutamatergic pathway activity in the NTS is due to the recruitment of inhibitory GABAergic interneurons that culminate in the reduction in NTS neuron activity, probably modulating the efferent pathway that stimulates the locus coeruleus, whose neurons modulate both neocortical activity and the first synaptic contact of sensory-discriminative pathways from the anterolateral system in the dorsal horn of the spinal cord and in the spinal nucleus of the trigeminus nerve. The neural network discussed here seems to be a strong candidate for evaluating the anticonvulsive effects of the vagus nerve stimulation procedure used for the treatment of epilepsy refractory to pharmacological treatment with anticonvulsants.

As a limitation of this investigation, seizure severity was recorded only by Racine’s index. New approaches, such as electroencephalography, are needed to assess seizure severity in individuals. Concerning postictal antinociception, only the tail-flick test was used as a nociceptive procedure. However, although there are other suitable experimental procedures for recording hypoalgesia organised by supraspinal structures, such as the hot place test, the tail-flick test was chosen because this procedure recruits only spinal reflexes, avoiding the unexpected effect of cortical spreading postictal depression on pain thresholds. Finally, considering that cortical neurons and other brain nuclei are connected to brainstem structures, such as the periaqueductal grey matter^49,50^, a midbrain structure that is also reported to play a role in the organisation of postictal antinociception, we cannot rule out the unexpected effect of postictal depression on the activity of supraspinal nuclei of the endogenous pain modulatory descending system during the elaboration of postictal antinociception. An example of the relevance of these nuclei is the critical role played by norepinephrinergic neurons of the locus coeruleus in the organisation of postictal antinociception, which was demonstrated by our team.

The excitotoxicity caused by increased Ca^++^ influx in neuronal cells is one of the primary mechanisms of cell loss in several diseases of the central nervous system. Ca^++^ influx-triggered excitotoxicity through Ca^++^-permeable AMPA glutamatergic receptors has been detected in multiple disease models, including epilepsy. For this reason, specific blockade of the AMPA receptor might be a more plausible strategy for delaying the onset and progression of tonic‒clonic seizures with fewer side effects than blocking NMDA glutamatergic receptors^51^. We cannot rule out the possibility that the excessive release of glutamate from the axon terminals of seizing animals or the overactivation of NMDA receptors in brain tissue caused by GABAergic dysfunctions after peripheral treatment with PTZ did not occur in this study or that the anticonvulsant effect of NMDA antagonists was related to the decrease in excitotoxicity triggered by acute insult.

Finally, considering that the effects of brainstem manipulation on tail-flick latencies seem to be more consistent than on seizures, we should highlight the relevance of each neuropharmacological treatment on postictal antinociception organisation rather than in tonic‒clonic seizures control.

## Data Availability

The datasets generated or analysed during the current study are not publicly available due to the limited space in the USP data repository at the time of submission of the manuscript but are available from the corresponding author upon reasonable request.
